# Evaluation of sampling methods for genomic surveillance of SARS-CoV-2 variants in aircraft wastewater: advancing global early-warning systems for future pandemics

**DOI:** 10.3389/fmicb.2025.1717424

**Published:** 2025-11-27

**Authors:** Opeyemi U. Lawal, Valeria R. Parreira, Fozia Rizvi, Melinda Precious, Rebecca E. V. Anderson, Alyssa K. Overton, Jennifer J. Knapp, Brittany Maxwell, Steven Thomas, Marcos Zambrano, Chrystal Landgraff, Manon D. Fleury, Natalie C. Knox, Trevor C. Charles, Lawrence Goodridge

**Affiliations:** 1 School of the Environment, University of Windsor, Windsor, ON, Canada; 2 Great Lakes Institute for Environmental Research, University of Windsor, Windsor, ON, Canada; 3 Guelph Wastewater Epidemiology Laboratory for Public Health (GWELPH), University of Guelph, Guelph, ON, Canada; 4 Canadian Research Institute for Food Safety (CRIFS), Department of Food Science, University of Guelph, Guelph, ON, Canada; 5 Department of Biology, University of Waterloo, Waterloo, ON, Canada; 6 Greater Toronto Airports Authority, Toronto, ON, Canada; 7 National Microbiology Laboratory, Public Health Agency of Canada, Winnipeg, MB, Canada; 8 Department of Food Science, University of Guelph, Guelph, ON, Canada; 9 National Microbiology Laboratory, Public Health Agency of Canada, Guelph, ON, Canada; 10 Metagenom Bio Life Science Inc., Waterloo, ON, Canada

**Keywords:** SARS-CoV-2, variants of concern, autosampler, passive sampler, aircraft wastewater, RT-qPCR, metagenomics

## Abstract

**Background:**

Severe acute respiratory syndrome coronavirus 2 (SARS-CoV-2) is an ongoing threat to global health. Wastewater-based surveillance (WBS) has proven to be an important tool for tracking the dissemination of SARS-CoV-2 variants of concern (VOCs) in the community. In Canada, metagenomic analysis of aircraft wastewater was adopted at an early stage of the pandemic to track importation of emerging variants into the country. However, the acute need to determine the presence of emerging SARS-CoV-2 sublineages meant that the sampling methods utilized were not adequately validated. Here, we compared two different sampling methods for genomic surveillance of SARS-CoV-2 VOCs in aircraft sewage samples.

**Methods:**

Eighty-eight composite wastewater samples were collected over 9 weeks using both autosampler and passive torpedo samplers at the same location. SARS-CoV-2 nucleic acid in the samples was quantified using RT-qPCR. RNA samples were extracted and sequenced with the MiniSeq system using the tiled-amplicon sequencing approach with ARTIC V4.1 primer sets. Raw reads were preprocessed and SARS-CoV-2 mutations, variants lineages, and other sequence metrics from the two sampling methods were compared.

**Results:**

The two sampling methods yielded comparable viral load by RT-qPCR, but the autosampler produced higher genome coverage relative to the passive samplers. The Omicron lineages identified differed by sampling method. BQ.1* and BA.5.2*, which were the predominant lineages in wastewater and clinical samples at the time, were identified as dominant in the autosampler and passive sampler, respectively. Additionally, the autosampler captured higher diversity and relative abundance of VOCs, including emerging variants (XBB* and CH.1* lineages), as well as more clinically relevant mutations (S:K444T, T22942A, S:R346T) relative to passive sampler. Overall, the passive samplers produced concordant results with the autosampler for measuring SARS-CoV-2 load with RT-qPCR in aircraft wastewater.

**Conclusion:**

Taken together, our results suggest underestimation of the diversity and abundance of SARS-CoV-2 VOCs and mutations in aircraft sewage using passive torpedo samplers. These data can be used to optimize genomic surveillance approaches for SARS-CoV-2 VOCs in aircraft wastewater samples.

## Background

The emergence and rapid spread of severe acute respiratory syndrome coronavirus 2 (SARS-CoV-2) is an ongoing threat to public health (da Silva Filipe et al., 2021; Aggarwal et al., 2022). Since the report of the first case in China in 2019, approximately 800 million people have been infected resulting in more than 7 million deaths globally (World Health Organization, 2024). The trajectory of this pandemic has been enhanced by increased community transmission and global travel that contributed to new and multiple introductions of variants into different regions and countries (Alm et al., 2020; da Silva Filipe et al., 2021; Guo et al., 2022). To minimize community transmission of known and emerging SARS-CoV-2 variants, global response measures were developed, including face mask mandates, social distancing, border closures, increased community testing, and negative test requirements at ports of entry (Shiraef et al., 2022; Tegally et al., 2022) among others. While these measures were effective during the early stages of the pandemic, they were seemingly unsustainable, particularly for stopping early community transmission (Peeling et al., 2022; Alvarez et al., 2023). Wastewater-based surveillance (WBS) has emerged as an important tool for measuring burden of disease and for tracking SARS-CoV-2 to provide early warning signals of community transmission of the virus (Ahmed et al., 2020; Landgraff et al., 2021; Arts et al., 2022; Bitter et al., 2022; Lawal et al., 2022; Oloye et al., 2022; Overton et al., 2024). This approach has proven effective in detecting the virus in communities, including in areas where clinical testing is limited or unavailable, and has provided early warning of outbreaks. WBS has also been used to evaluate the effectiveness of public health interventions over time (Bitter et al., 2022; Hubert et al., 2022; Lawal et al., 2022; Oloye et al., 2022; Overton et al., 2024; Thakali et al., 2024).

Three main sampling methods are employed during WBS, including autosampling, passive sampling, and grab sampling (Reeves et al., 2021; Schang et al., 2021; Habtewold et al., 2022; Wilson et al., 2022). Grab sampling, which entails collection of a sample at a single point in time, is limited by its inability to capture temporal variations in contaminant levels, leading to potentially unrepresentative snapshots of the overall pollution load, and it may miss transient events or spikes in contaminant concentrations (Cornman et al., 2018). Autosamplers employ a peristaltic pump to automatically collect wastewater samples (Wilson et al., 2022). This device is commonly used for the collection of grab or composite samples and time-weighted average samples (Wilson et al., 2022). However, the major limitation of this device is its high cost, and the maintenance required to ensure accurate and reliable operation. Additionally, if the sampling site is in a busy thoroughfare such as a road or a parking lot, it may not be possible to install an autosampler. Alternatively, passive samplers are simple, cost-effective devices that can be deployed in various locations within the wastewater system, including influent, effluent, and within the wastewater treatment plant for an extended period (Schang et al., 2021). The passive sampler method involves the use of a porous membrane or sorbent material that captures and concentrates the target analytes in the wastewater (Schang et al., 2021). Both passive and autosamplers can collect samples over a set time. The main advantages of passive samplers are their simplicity and low cost, lack of power or specialized infrastructure requirement, making them an attractive option for large-scale deployment in communities, in addition to providing a representative sample of the wastewater composition over time, enabling the detection of low levels of the target analytes (Schang et al., 2021; Habtewold et al., 2022).

However, the main limitation of passive sampling is the inability to collect frequent (timed collection of set volumes, so cannot collect a controlled composite sample) samples, which may limit their effectiveness in tracking changes in wastewater composition over time (Bivins et al., 2022).

The effectiveness of different sampling approaches for the quantification of SARS-CoV-2 in wastewater have been assessed with different conclusions. For example, Habtewold et al. (2022) employed RT-qPCR to evaluate three passive sampler materials (electronegative membranes, gauzes, and cotton buds) for their ability to concentrate and quantify SARS-CoV-2 in wastewater in comparison to composite samples from an autosampler, and reported that after 24-h of deployment (a common timeframe for collecting wastewater samples using autosamplers), the electronegative membranes and gauzes had 8/9 samples positive for SARS-CoV-2 RNA, while only 6/9 composite samples collected by autosampler were positive. Schang et al. (2021) reported similar results where they observed greater sensitivity of the passive samplers than liquid composite autosampler. Other studies (Ahmed et al., 2020; Wu et al., 2020; Wilson et al., 2022) have shown that autosampling and passive sampling methods were effective in detecting SARS-CoV-2 in wastewater, with comparable levels of sensitivity. While autosampling was found to be more effective in detecting the virus in wastewater with low viral concentrations, passive sampling was more sensitive in samples with high viral loads because passive sampling was reported to be affected by interference from various compounds in the wastewater, which could impact the accuracy of the results (Bivins et al., 2022).

It is noteworthy that all currently available studies comparing auto- and passive sampling for the surveillance of SARS-CoV-2 in wastewater used quantitative reverse transcription PCR (RT-qPCR)-based assays that target the amplification of a small fragment (~70 bp) of the SARS-CoV-2 genome (Lu et al., 2020; Choudhury et al., 2021). Hence, it is not clear how auto and passive sampling would compare with respect to metagenomic studies, including the reconstruction of the whole SARS-CoV-2 genome, and the characterization of mutation signatures of variants of concern (VOCs) in wastewater. Here, we evaluated 24-h composite samples obtained from autosamplers, and passive torpedo samplers on the same days (Schang et al., 2021; Habtewold et al., 2022) for genomic surveillance of SARS-CoV-2 VOCs in aircraft wastewater samples. We assessed the viral load, SARS-CoV-2 genome completeness, and the relative abundance and diversity of SARS-CoV-2 lineages, as well as clinically relevant mutations in the wastewater samples.

## Materials and methods

### Wastewater sample collection

Samples consisting of pooled wastewater from aircraft lavatory waste were obtained from the onsite triturator building at Pearson International Airport in Mississauga, Ontario, Canada. Samples were collected 5 days per week for 9 weeks for a total of 88 samples (each day, one 24 h composite sample was collected from the autosampler, and a corresponding 24 h sample was collected from the passive sampler) between November 2022 and January 2023, using torpedo passive samplers (one per sample) and an autosampler (Hach AS950 portable compact sampler). These samples represent a subset of samples collected as part of the wastewater-based genomic surveillance initiative for tracking SARS-CoV-2 VOCs at Pearson International Airport (Arts et al., 2022; Global News, 2024). Torpedo passive samplers were adopted because of their efficiency, portability, cost-effectiveness, and ease of deployment (Schang et al., 2021), while the autosampler was used for its uniformity with other sampling programs and ease of access to units during the COVID-19 supply chain interruption. All samples were collected under the same conditions and maintained on ice during transport to the Canadian Research Institute for Food Safety on the campus of the University of Guelph, where they were processed within 24 h.

### Sample preparation and total nucleic acid (tNA) extraction

Wastewater samples collected with passive samplers were submitted to the laboratory as intact passive samplers containing four gauze squares (10 cm × 10 cm). One of the gauzes was placed into a filtered stomacher bag containing 50 mL of sterile PBS with 0.05% Tween 80 and an antifoaming agent (Thermo Fisher, Burlington, ON) and stomached at 200 rpm for 2 min. tNA was extracted from the recovered filtrate. Since samples from the autosampler were already in liquid form, tNA was extracted directly from these samples without a preprocessing step using Nanotrap magnetic virus particles (Ceres Nanosciences, Manassas, VA, USA) as described (Lawal et al., 2022). Briefly, 600 uL of Nanotrap particles were added to 50 mL of each wastewater sample and incubated at room temperature on a rotator platform at 100 rpm for 20 min. Following incubation, the Nanotrap particles were concentrated away from the wastewater using a DynaMag-2 magnet rack at 4 °C (Thermo Fisher, Burlington, ON), and the wastewater was discarded. The Nanotrap particles (with attached virus particles) were subjected to tNA extraction using the QIAamp Viral RNA extraction Mini kit (QIAGEN, Louisville, KY, USA) and the automated QIAcube instrument (QIAGEN) according to the manufacturer’s instructions.

### Reverse transcriptase-real time quantitative PCR (RT-qPCR)

The tNA samples were subjected to one-step reverse transcriptase RT-qPCR using primers/probe mix for detection of the N1 and N2 regions with the 2019-nCoV CDC RUO kit (IDT, Coralville, IA, USA) and TaqPath ready-to-use master mix (Thermo Fisher), as described by the US Center for Disease Control and Prevention design (Centers for Disease Control and Prevention (CDC), 2023). RT-qPCR was performed on a QuantStudio 5 instrument (Applied Biosystems, Thermo Fisher) as described previously (Lawal et al., 2022). Wastewater samples with a cycle threshold (Ct) of ≤36 were sequenced.

### Amplicon preparation and sequencing

Complementary DNA (cDNA) was generated using the SuperScript™ IV First-Strand Synthesis System (Thermo Fisher) according to the manufacturer’s specifications and as previously described (Lawal et al., 2022). The entire length of the SARS-CoV-2 genome was amplified using tiled amplicon PCR reactions performed on an Eppendorf Mastercycler X50a (Eppendorf, Mississauga, ON) using ARTIC V4.1 primers (IDT; 10008554) and Q5 Hot Start Master mix (New England Biolabs, Whitby, ON) The amplification parameters, cleanup, and the amplicon quantification protocol were performed as previously described (Lawal et al., 2022).

### Library preparation and tiled-amplicon sequencing

Amplicon libraries were prepared using the Nextera XT DNA library prep kit (Illumina, Harrisburg, PA, USA) with dual-index barcodes following manufacturer’s instructions. Paired-end (2 × 150 bp) sequencing of the libraries was performed on Illumina MiniSeq systems using the tiled-amplicon sequences that were generated with ARTIC V4.1 primers.

### Bioinformatics analysis

Raw reads were analyzed using the ViralRecon pipeline v2.4.1 (Patel et al., 2021). SARS-CoV-2 variants were called with iVar (Grubaugh et al., 2019) using a minimum frequency of 0.01 and quality score threshold of 15. Consensus sequences were generated with a minimum frequency, quality score, and read depth of ≥50%, 15, and 20, respectively. Variant clades and lineages from the consensus sequences were analyzed using Nextclade v2.10 (Aksamentov et al., 2021), and Pangolin v4.2 (O’Toole et al., 2021), respectively. The relative abundance of SARS-CoV-2 lineages and the frequencies of diagnostic and clinically important mutations associated with VOCs that were present were assessed using Alcov (Ellmen et al., 2021).

### Statistical analysis

Normality of the differences observed between methods were assessed and tested using the Shapiro–Wilk test. In the case where normality was not met, the non-parametric Wilcoxon Signed-Rank test was used for analysis to compare the two methods. The Pearson’s correlation coefficient was calculated to assess correlation between different measure types. All statistical analyses were conducted using R software v4.4.1 (R Core Team, 2024). The differences were deemed statistically significant with a *p*-value <0.05.

### Data availability

The metagenomic sequences are available in the NCBI Sequence Read Archive under submission accession number PRJNA1238906.

## Results

### Autosampling and passive sampling detected largely consistent viral loads in aircraft wastewater samples

**Figure 1:**
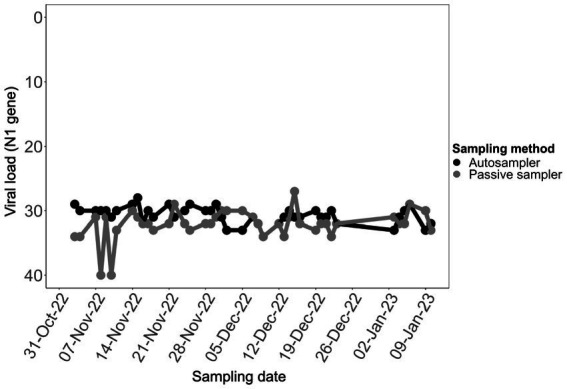
Comparison of viral loads in aircraft sewage samples collected with different sampling methods. Samples collected with autosampler (black), and passive sampler (gray) contained relatively comparable viral load (*p* < 0.05).

The viral load of SARS-CoV-2 in aircraft wastewater samples that were collected using the two different sampling methods was based on the quantification of the N1 region as determined by RT-qPCR. Aircraft triturator wastewater samples had high viral loads with Ct values ranging between 27 and 34, except for two samples collected with passive sampler in the first 2 weeks of the study period, which had Ct values of 40. Ct values for composite samples collected by autosampling within the first 3 weeks of the study averaged 30, whereas samples from passive sampling yielded higher Ct values corresponding to lower viral loads (*p* < 0.05) (Figure 1). In a unique instance, the viral load in a sample collected using a passive sampler on December 15, 2022, was slightly higher than that in a sample collected using an autosampler on the same date (Supplementary Table 1 and Figure 1). Overall, the viral loads in samples from both sampling methods were largely consistent over time.

### Autosampling produced higher SARS-CoV-2 genome coverage and completeness

**Figure 2:**
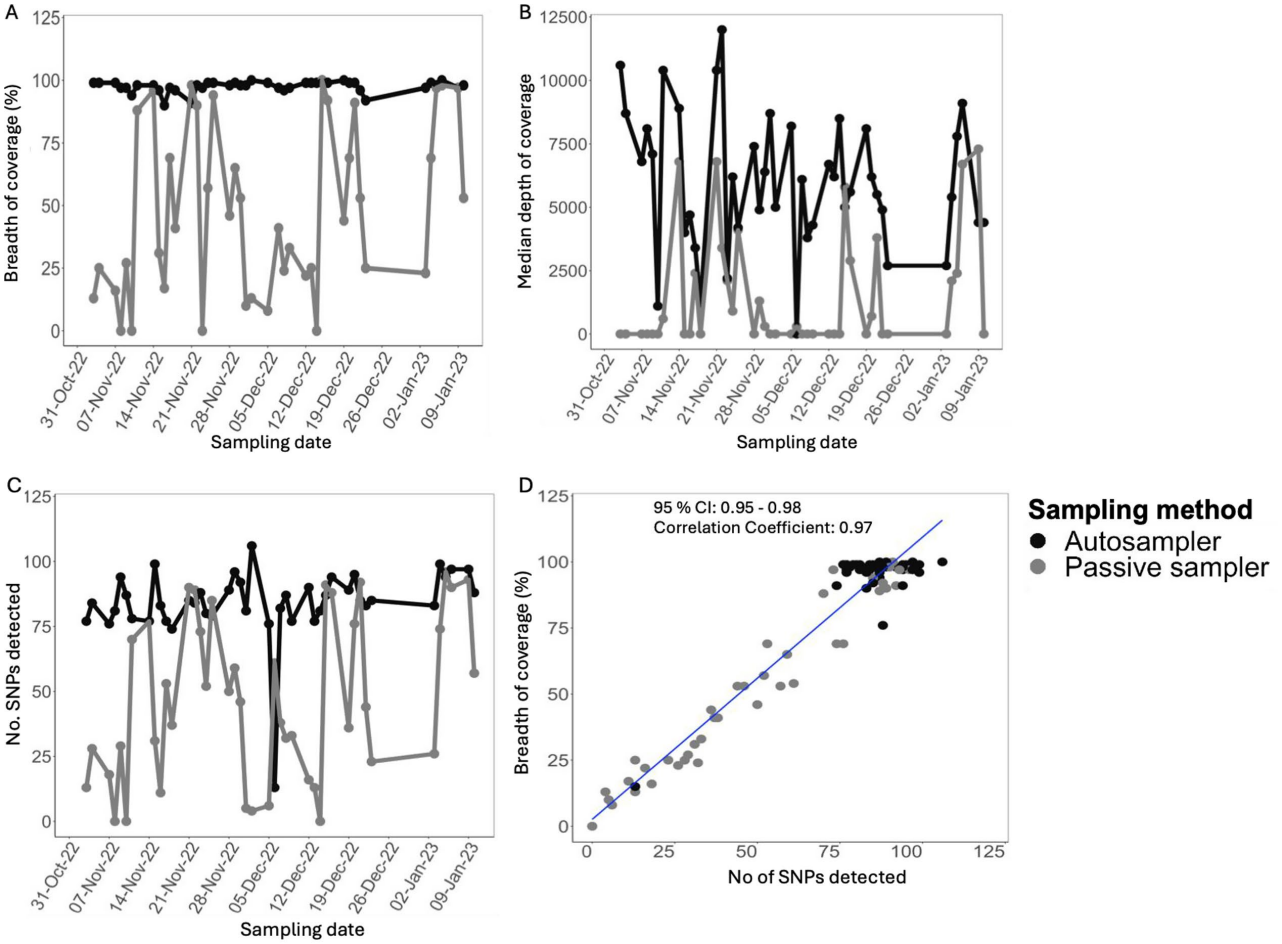
Comparison of sequence metrics for aircraft sewage samples collected using different sampling methods over 9 weeks **(A)**. Measure of genome completeness **(B)**. Overall genome median depth of coverage **(C)** Comparison of total single nucleotide polymorphisms (SNPs) detected in the samples. **(D)** Pearson’s correlation between genome completeness and number of SNPs detected in aircraft sewage samples.

Overall, 85/88 samples were sequenced. Three samples collected with the passive sampler had high Ct value (>36) or failed at library preparation stage and were not sequenced (Supplementary Table 1). A total of 250,665,771 mapped reads were obtained from the samples that were sequenced (mean, 2,949,009; range, 325,488–6,203,127 per sample). Specifically, the autosampler yielded 127,887,127 mapped reads, while passive sampling yielded 11,142,372 fewer reads. (Supplementary Table 1). The breadth of coverage of SARS-CoV-2 of the consensus sequences, which measures genome completeness relative to the reference SARS-CoV-2 Wuhan strain, was consistently higher in samples from the autosampler, ranging between 90 and 100% in the great majority (*n* = 42/44; 95%) of the samples over the study period. In contrast, passive samplers yielded lower genome completeness relative to corresponding autosampler samples (Figure 2A). A similar trend was observed for the median depth of coverage, which measures the overall median number of reads aligning to each position in the reference genome (Figure 2B). Likewise, the overall total single nucleotide polymorphisms (SNPs) detected in samples from passive sampling were lower relative to those detected in autosampler samples (Figure 2C). Notably, the breadth of coverage strongly correlated with the overall number of SNPs covered and detected in each sample (Pearson’s Correlation Coefficient = 0.97; 95% confidence interval: 0.95–0.98) (Figure 2D). Overall, sequence metrics from samples collected with the autosampler indicated higher quality compared to corresponding samples from passive sampling.

### Differential SARS-CoV-2 VOCs abundance and diversity in aircraft sewage samples collected with different sampling methods

**Figure 3:**
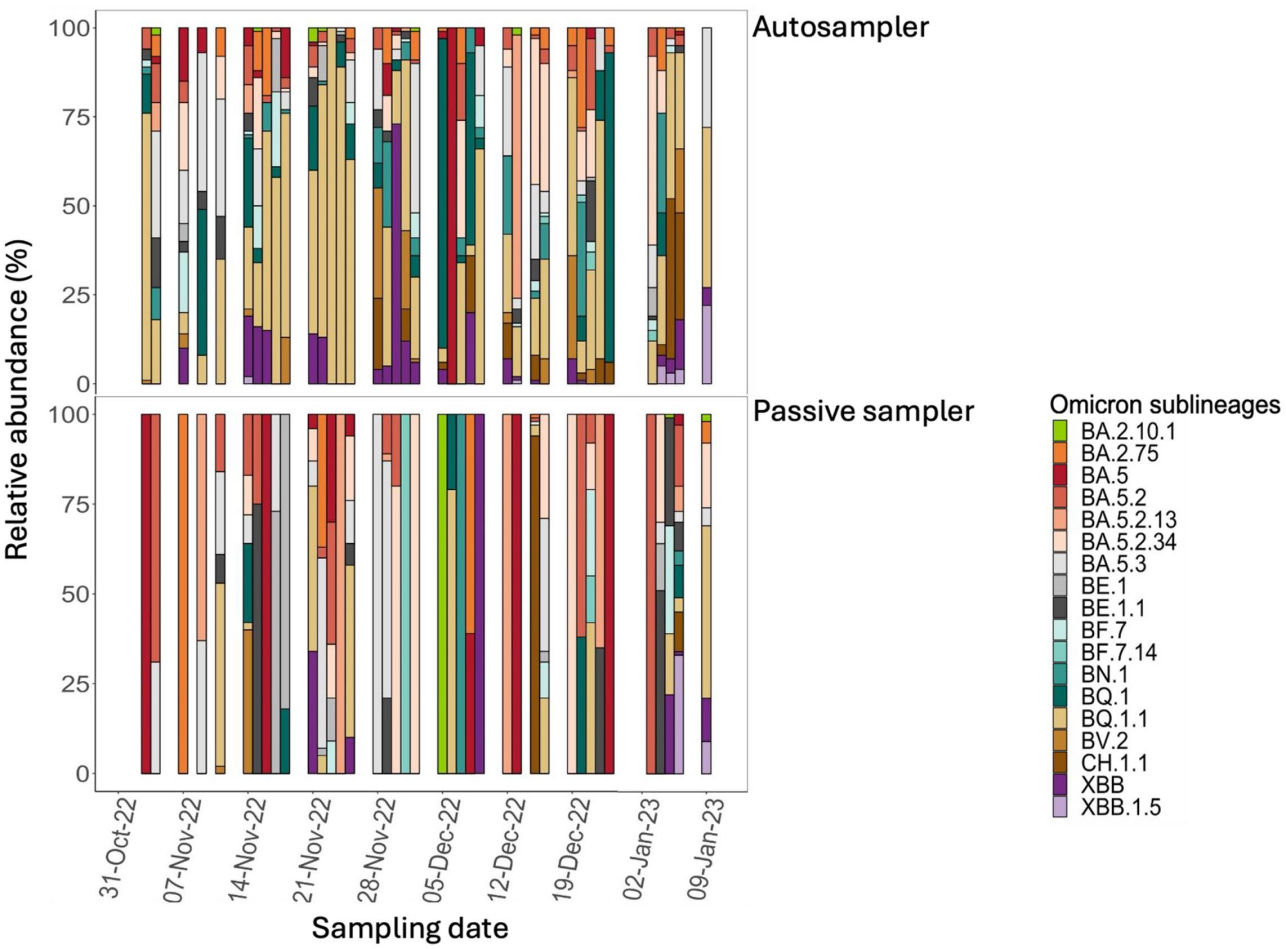
Comparison of SARS-CoV-2 variant diversity in aircraft sewage samples collected using two sampling methods over 9 weeks. Aircraft sewage samples were collected using an autosampler (upper figure), with corresponding samples collected using a passive sampler (lower panel) over 9 weeks. Sequence reads were mapped to the Wuhan reference strain, and SARS-CoV-2 lineages were identified using the Alcov pipeline. Different colors represent distinct viral lineages, showing variations in lineage composition detected by each sampling method.

To assess the dominant SARS-CoV-2 lineage in samples collected with autosamplers and passive samplers, we analyzed the consensus sequences using Pangolin and Nextclade (see *Methods*). The detected SARS-CoV-2 lineages during the study period included BQ.1* (*n* = 23/85, 27%), BA.5.2* (*n* = 17/85, 20%), and other BA.5 sublineages, with emerging VOCs XBB* and CH.1* observed in four samples (Supplementary Table 1). Dominant lineages varied depending on the sampling method, with BQ.1* (*n* = 19/44, 43%) being predominant in autosamples, while BA.5.2* (*n* = 12/41, 29%) dominated in consensus sequences of samples from passive samplers (Supplementary Table 1). Although both sampling methods captured Omicron as the dominant lineage, the identified Omicron sublineages differed, suggesting that the sampling method could influence the detection of dominant SARS-CoV-2 variant sublineages in wastewater. In addition to comparing dominant SARS-CoV-2 lineages/sublineages between autosampler and passive samplers, we assessed the relative abundance and differential diversity of all detected lineages (Ellmen et al., 2021). On average, autosampler samples contained seven different SARS-CoV-2 lineages per sample, while passive samplers had three lineages per sample (Figure 3). There were instances where autosampler samples contained up to 10 SARS-CoV-2 lineages, while only a few were detected using the passive sampler (Supplementary Figure 1). For example, all samples collected in the first week of November 2022 contained between 7 and 10 variant lineages, whereas the passive sampler detected two or fewer in the same samples. Notably, during this period, the variant lineages identified by the autosampler included circulating lineages (such as the BQ* and BE* lineages), which were not detected by the passive sampler at that time (Figure 3). Overall, autosamplers captured a higher diversity of SARS-CoV-2 lineages compared to passive samplers, including minor lineages and emerging VOCs (*p*-value < 0.05). The autosampler captured the emerging SARS-CoV-2 lineages earlier than the passive sampler. For example, the autosampler detected traces of the emerging XBB* lineage in the first week of November 2022, shortly after its first detection in Canada in the last week of September 2022, and at a time when only six cases had been reported in Canada (CovSPECTRUM, 2024). The autosampler consistently detected this variant lineage in the following weeks, whereas it was only detected in the passive sampler during the third week of November. Conversely, the BF.7* lineage detected in passive sampler samples was only identified in trace amounts in autosampler samples (Figure 3). This trend of earlier detection in autosampler samples persisted throughout the study period, indicating that autosamplers captured a greater diversity of SARS-CoV-2 lineages in aircraft wastewater samples than passive samplers.

### Autosampling captured more and higher frequencies of SARS-CoV-2 diagnostic mutations in aircraft wastewater

**Figure 4:**
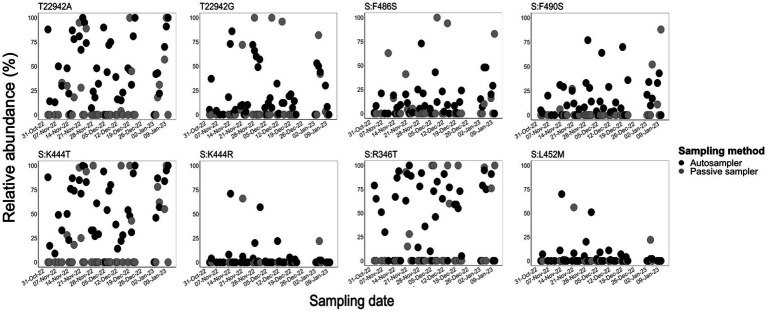
Comparative abundance of SARS-CoV-2 variants of concern (VOC)-diagnostic and alert mutations in aircraft sewage samples by sampling methods. These scatter plots show the relative abundance of VOC-diagnostic and alert mutations in aircraft sewage samples collected using autosampler (black) and passive samplers (gray). Sequence reads were mapped to the Wuhan reference strain, and selected mutations were quantified using the Alcov pipeline. Autosampling captured higher abundances of VOC diagnostic mutations and clinically significant mutations compared to passive sampling.

We assessed the efficiency of sampling methods in capturing diagnostic and/or clinically significant mutations in the spike protein of SARS-CoV-2 detected in aircraft wastewater samples by determining the relative frequency of eight selected mutations using Alcov (Ellmen et al., 2021). These mutations, including the spike protein mutations N460K (T22942A, T22942G), F486S, F490S, K444T, K444R, and L452M, as well as the FLiRT mutations (S:R346T, and S:F456L), which are reported to mediate immune evasion or escape from monoclonal and vaccine-induced antibodies (Tegally et al., 2022; Wang et al., 2022; Chakraborty et al., 2023; Planas et al., 2023; Zappa et al., 2023; Li et al., 2024; Overton et al., 2024). In comparison to passive samplers, the autosampler consistently captured these mutations (Figure 4). For example, the T22942A and T22942G mutations, which encode the N460K amino acid change in the spike protein, were detected in 77% (T22942G: *n* = 34/44) and 89% (T22942A: *n* = 39/44) of autosampler samples, with relative abundances ranging from 1 to 100%. In contrast, these mutations were detected in only 22% (*n* = 9/41) and 32% (*n* = 13/41) of passive sampler samples, with relative abundances between 4 and 100%. Similarly, the FLiRT mutation S:R346T was found in over 70% of autosampler samples, but in only 27% of passive sampler samples (Figure 4; Supplementary Figure 2). Overall, the autosampler consistently detected these mutations in a higher proportion of samples and at moderately higher frequencies compared to the passive sampler (*p* < 0.001) (Figure 4; Supplementary Figure 2).

## Discussion

We present a comparative analysis of two sampling methods, autosampling, and passive sampling, for genomic surveillance of SARS-CoV-2 lineages in pooled aircraft wastewater. We assessed both sampling methods for effectiveness in estimating the viral RNA concentration, relative abundance of clinically significant mutations in the samples, as well as comparing the quality of the reconstructed SARS-CoV-2 genomes obtained from tiled-amplicon sequencing.

The viral load in the aircraft sewage samples was high and largely comparable with both sampling methods during the study period. This observation aligns with previous studies that quantified SARS-CoV-2 RNA in wastewater by RT-qPCR, which found no significant differences between viral loads observed with autosampling and passive sampling (Ahmed et al., 2020; Wu et al., 2020; Habtewold et al., 2022; Wilson et al., 2022). However, amplicon-based sequencing of the samples revealed differences in the breadth of coverage and the total number of SNPs detected between the two sampling methods. Of note, samples were collected, maintained, and transported under the same condition, hence the difference could not have been due to sample handling. Since our passive sampling approach concentrated viral RNA in gauze, there may have been accumulation of RNA-degrading compounds in the matrix, which could lead to reduced or no amplification or negatively impacting the recovery of larger RNA fragments suitable for 400-bp amplicon sequencing (Bayati et al., 2022; Shakallis et al., 2022). This may not be apparent with RT-qPCR, as smaller fragments (~70 bp) are required for amplification (Lu et al., 2020; Choudhury et al., 2021).

Pooled aircraft wastewater, as sampled from a triturator, differs in characteristics from influent obtained from a wastewater treatment plant. For example, upon completion of the draining process from aircraft tanks, a solution containing disinfectant and deodorizer (commonly known as “blue juice” and available under various commercial names) is injected into each waste tank to minimize the inherent sanitary and odor problems associated with storing raw sewage (Lacey et al., 2010; Bivins et al., 2024). Typically, blue juice is a sodium hydroxide (caustic soda) solution, which likely degrades viral RNA. Following rinsing, the tanks may be primed with a small amount of disinfectant before receiving wastewater during subsequent flight segments. The wastewater accumulated in lavatory service trucks is then discharged into the municipal wastewater collection system through the triturator (Lacey et al., 2010; Bivins et al., 2024). Thus, the presence of concentrated disinfectants (including blue juice) in triturator samples, may lead to instability and degradation of viral RNA, especially in passive samples in which gauze is used, where these chemicals are further concentrated.

While consensus sequences provides information on the dominant SARS-CoV-2 lineage, the relative abundance of all lineages in wastewater allows for tracking emerging variants and mutations, and the replacement of known lineages with emerging ones in the community (Ellmen et al., 2021; Fontenele et al., 2021, 2023; Lawal et al., 2022; Oloye et al., 2022; Overton et al., 2024). While several computational pipelines are available for assessing SARS-CoV-2 variants in wastewater. Alcov, used in this study, has been shown to perform on par with leading tools, demonstrating robust accuracy and reliability (Sutcliffe et al., 2024).

Our results showed variations in the dominant SARS-CoV-2 lineages captured by each sampling method. BQ.1* (BQ.1 and BQ.1.1), the main lineage circulating in local communities, and more broadly, the province of Ontario during the study period (Public Health Ontario, 2022; Akash et al., 2023) was dominant in aircraft sewage samples collected with the autosampler but was less frequently captured with passive sampling. Likewise, mutations in the SARS-CoV-2 spike protein, which are crucial for host immune invasion and antibody and vaccine evasion (Tegally et al., 2022; Wang et al., 2022; Chakraborty et al., 2023; Planas et al., 2023; Zappa et al., 2023; Li et al., 2024) were captured at significantly higher frequencies with autosampling relative to passive sampling, this suggests that passive sampling might underestimate the presence and relative abundance of these mutations in wastewater. We were unable to compare these findings with existing studies, as, to the best of our knowledge, no studies specifically assess or compare these sampling methods for their effectiveness in capturing clinically significant mutations.

It is important to note a limitation of this study. For example, we opted for gauze as the sampling material in the passive sampler based on previous work from our group, demonstrating its high efficiency in capturing SARS-CoV-2 in wastewater (Habtewold et al., 2022). The torpedo sampler used in our study can also accommodate additional sampling materials such as electronegative membranes and cotton buds. It is plausible that using these alternative materials could have resulted in more comparable results with the autosamplers. Nevertheless, in this study, we restricted our choice of materials since samples were being collected as part of the ongoing surveillance program. Introducing a greater diversity of materials within the passive sampler would have substantially increased the number of samples needing processing, thereby delaying result reporting, and adding extra financial costs.

In conclusion, WBS is a valuable tool for genomic surveillance of SARS-CoV-2 in aircraft wastewater. Passive samplers produce concordant results with autosamplers for detection and quantification of SARS-CoV-2 with RT-qPCR, but for genomic detection of Omicron sublineages, autosamplers were more effective, providing better genome coverage and higher efficiency in capturing lineages and diagnostic mutations at greater relative abundance. Our results demonstrate that autosampler composites yielded higher SARS-CoV-2 genome completeness and variant resolution, supporting their use for continuous genomic surveillance in facilities with reliable infrastructure. Passive samplers, while prone to partial RNA degradation, provide a practical alternative for resource-limited or remote sites where power and maintenance are constraints. Integrating both approaches can optimize surveillance coverage and resilience across diverse settings and pathogens. This data can be used to optimize genomic surveillance approaches for SARS-CoV-2 lineages in wastewater. Further research is needed to evaluate the effectiveness of both methods under different conditions and with different sublineages to identify potential improvements to enhance the accuracy and reliability of WBS of SARS-CoV-2 in aircraft wastewater.

## Data Availability

The metagenomic sequences are available in the NCBI Sequence Read Archive under submission accession number contains an hyperlink for the sequence data accession number PRJNA1238906.
